# Isolated Neuropathic Bladder Associated With Human T-lymphotropic Virus Type 1 Infection Precipitating Acute Focal Bacterial Nephritis: A Case Report

**DOI:** 10.7759/cureus.81079

**Published:** 2025-03-24

**Authors:** Takahiro Minamii, Takahiro Nakajima, Shinpei Mizuki, Yohei Kanzawa, Naoto Ishimaru

**Affiliations:** 1 General Internal Medicine, Kobe City Medical Center General Hospital, Kobe, JPN; 2 General Internal Medicine, Akashi Medical Center Hospital, Akashi, JPN

**Keywords:** acute focal bacterial nephritis, acute kidney injury, acute pyelonephritis, htlv-1, neurogenic bladder, neuropathic bladder

## Abstract

A 71-year-old woman with previously undiagnosed human T-lymphotropic virus type 1 (HTLV-1) infection presented with fever, urinary difficulties, acute abdominal pain, and severe acute kidney injury (AKI). Diagnostic evaluations confirmed acute pyelonephritis (APN) and acute focal bacterial nephritis (AFBN). Despite antibiotic treatment, she required temporary hemodialysis and eventually developed chronic kidney disease. This case highlights the importance of considering APN and AFBN as differential diagnoses in patients presenting with acute abdominal pain and severe AKI. It also underscores the need to investigate predisposing conditions. Notably, neuropathic bladder can be an isolated neurogenic manifestation of chronic HTLV-1 infection. Therefore, a thorough assessment of urinary dysfunction is essential in patients with APN and chronic HTLV-1 infection.

## Introduction

Human T-lymphotropic virus type 1 (HTLV-1) is a human retrovirus infecting approximately 20 million people worldwide [1]. The infection has a distinct geographic distribution, with high prevalence observed in regions such as Japan, Africa, the Caribbean islands, and Central and South America [2]. According to epidemiological data from blood donor screening, the Kyushu-Okinawa area is considered the most endemic region for HTLV-1 infection in Japan [2].

Infected individuals typically remain asymptomatic, but the virus can eventually cause adult T-cell leukemia and HTLV-1-associated myelopathy (HAM). HAM is a rare complication of chronic HTLV-1 infection, occurring in approximately 0.25% of HTLV-1 carriers in Japan and 1.9-3.7% in other countries [3-6]. It is characterized by progressive spastic paraparesis primarily affecting the lower limbs with an insidious onset, often accompanied by autonomic symptoms such as neuropathic bladder [7].

Chronic HTLV-1 infection has been associated with neuropathic bladder, even in the absence of other neurological abnormalities [8]. Neuropathic bladder is a recognized risk factor for UTIs [9]. Here, we present a case of acute pyelonephritis (APN) and acute focal bacterial nephritis (AFBN), manifesting as acute abdominal pain and severe acute kidney injury (AKI), precipitated by a neuropathic bladder secondary to chronic HTLV-1 infection.

## Case presentation

A 71-year-old Japanese woman was admitted to our hospital with a five-day history of fever and lower abdominal pain. The day before admission, she developed severe, sharp, and constant lower abdominal pain.

The patient reported experiencing urinary difficulties over several years, with a medical history that included multiple episodes of UTI. She had consulted a urologist on several occasions and received antibiotic therapy for each episode. She was not taking any medications or nutritional supplements.

On physical examination, her blood pressure was 100/71 mmHg, heart rate was 90 bpm, respiratory rate was 27 breaths/min while breathing ambient air, and body temperature was 36.0°C. She was conscious and alert. Severe generalized abdominal pain radiating from the lower abdomen with guarding was noted. Costovertebral angle tenderness was present bilaterally. Other findings, including the neurologic examination, were unremarkable.

Her laboratory findings are shown in Table 1.

**Table 1:** Summary of laboratory investigations ANA, anti-nuclear antibody; ANCA, anti-neutrophil cytoplasm antibody; ASO, anti-streptolysin O; BUN, blood urea nitrogen; GBM, glomerular basement membrane; HbA1c, hemoglobin A1c

Test	Result	Reference range
White blood cells	29,370/μL	3,300-86,000/μL
Hemoglobin	13.3 g/dL	11.6-14.8 g/dL
Platelets	66,000/μL	158,000-348,000/μL
BUN	97 mg/dL	8-20 mg/dL
Creatinine	5.41 mg/dL	0.46-0.79 mg/dL
Calcium	7.9 mg/dL	8.0-10.0 mg/dL
CRP	27.79 mg/L	0-0.14 mg/dL
HbA1c	6.30%	≥6.5% (4.9-6.5%)
ANA	Negative	Negative
Anti-DNA antibody	Negative	Negative
ANCA	Negative	Negative
Anti-GBM antibody	Negative	Negative
ASO antibody	Negative	Negative
Hepatitis C serology	Negative	Negative
Complement C3	94 mg/dL	73-138 mg/dL
Complement C4	19.1 mg/dL	11-31 mg/dL
CH50	38.9 U/mL	25-48 U/mL

The findings from the urinalysis are summarized in Table 2.

**Table 2:** Results of urinalysis FENa, fractional excretion of sodium; NAG, N-acetyl-β-D-glucosaminidase

Test	Result	Reference range
White blood cells	30-49/HPF	0-5/HPF
Red blood cells	>100/HPF (isomorphic)	0-3/HPF
NAG	49.5 IU/L	<11.5 IU/L
β2-Microglobulin	45.227 μg/L	<150 μg/L
Granular casts	Present	Absent
FENa	12.70%	>2% (renal/intrinsic)

**Figure 1:**
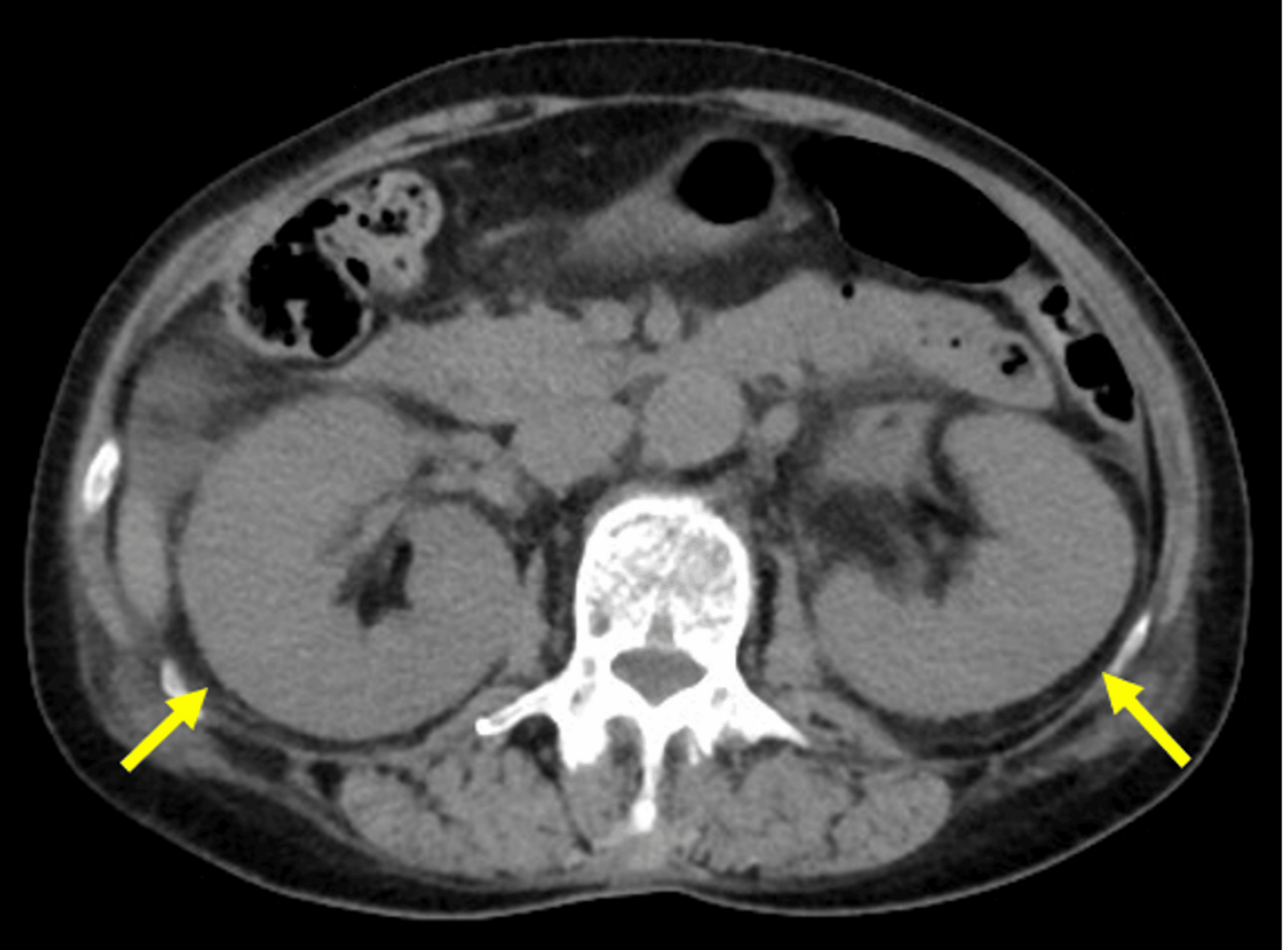
CT showing bilateral nephromegaly

Non-contrast CT images revealed bilateral renal enlargement and perinephric fat stranding without evidence of hydronephrosis or nephrolithiasis (Figure 1).

**Figure 2:**
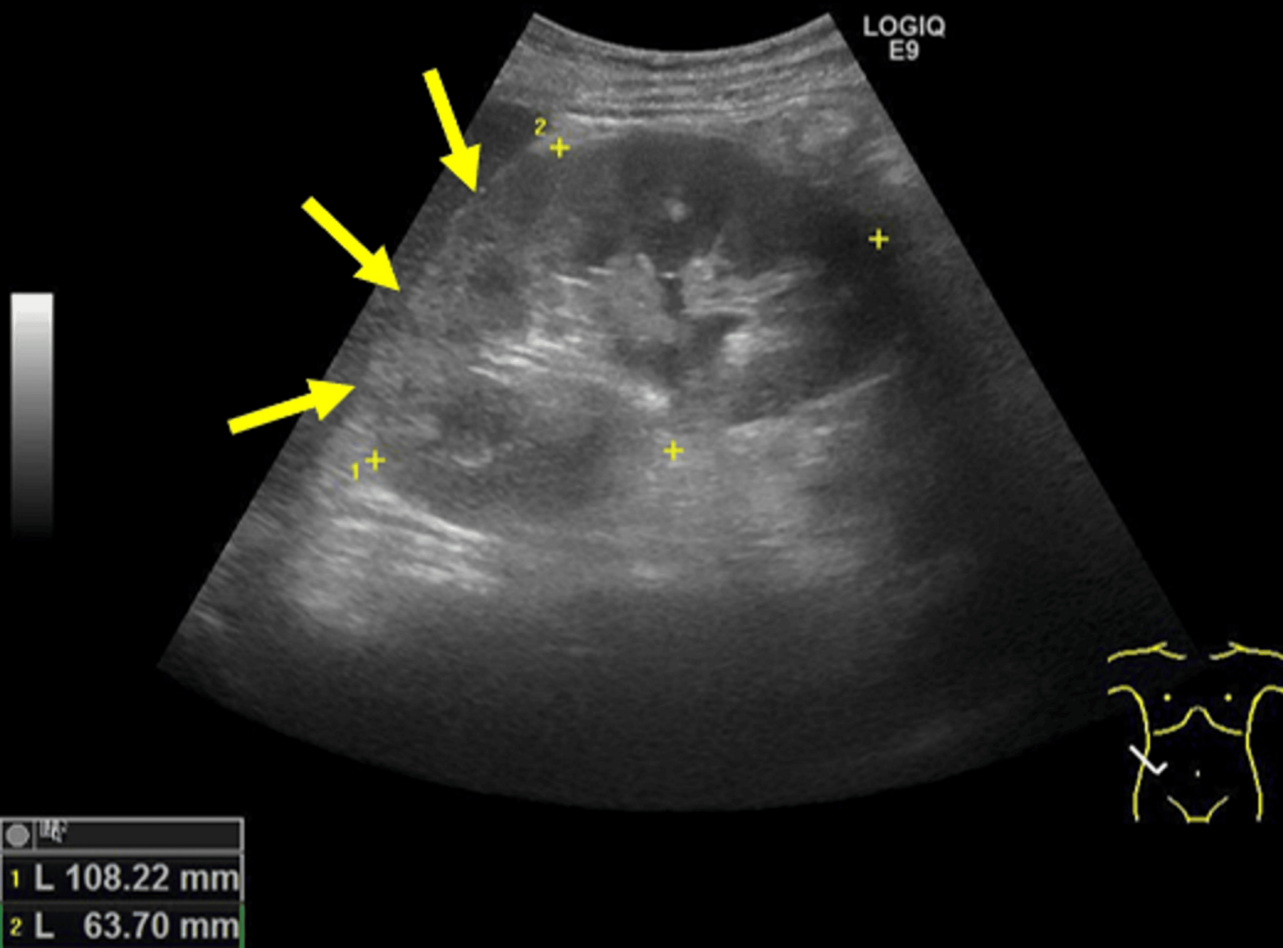
Color-Doppler-enhanced ultrasonography of the abdomen demonstrating a hyperechoic focal lesion in the right kidney

**Figure 3:**
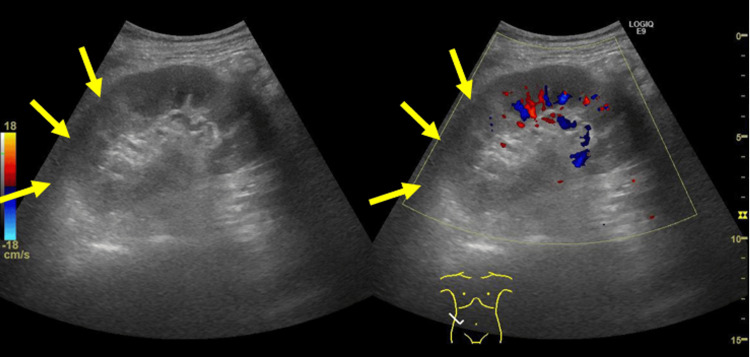
Hyperechoic focal lesion demonstrating decreased blood flow

The acute abdomen had no clear etiology, such as intestinal perforation or appendicitis. Color-Doppler-enhanced ultrasonography of the abdomen demonstrated bilateral renal enlargement and a hyperechoic focal lesion with decreased blood flow (Figure 2, Figure 3).

Blood and urine cultures were positive for *Escherichia coli*, which was susceptible to cefazolin, cefmetazole, and levofloxacin. Fever, pyuria, bacteriuria, and radiological findings suggested a diagnosis of APN, more specifically AFBN, with bloodstream infection. We initiated empiric therapy with IV cefmetazole at a dosage of 1 g/day, guided by the local antibiogram. Concurrently, we began aggressive hydration due to suspicion of pre-renal AKI. However, her urine output remained below 200 mL/day, and her creatinine level increased from 5.41 mg/dL to 7.38 mg/dL by the fourth day of admission, prompting us to start intermittent hemodialysis. After two sessions of hemodialysis, her urine output began to increase, and her creatinine level gradually improved, allowing us to discontinue hemodialysis.

**Figure 4:**
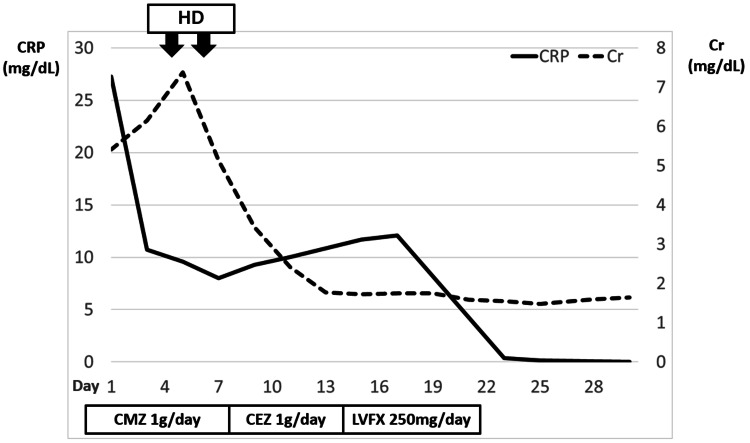
Clinical course of the patient

By the eighth day of admission, the patient became afebrile, and her abdominal pain resolved. The antibiotic was changed to IV cefazolin based on the susceptibility data of *E. coli *detected in the blood culture. On the 14th day of treatment, the patient developed cefazolin-induced thrombocytopenia, necessitating a modification of the antibiotic regimen. We discontinued cefazolin and initiated levofloxacin as an alternative antimicrobial agent, continuing therapy until the 21st day. This extended antibiotic course was implemented because prolonged treatment is typically required for AFBN compared to APN [10]. The patient’s clinical course is shown in Figure 4.

**Figure 5:**
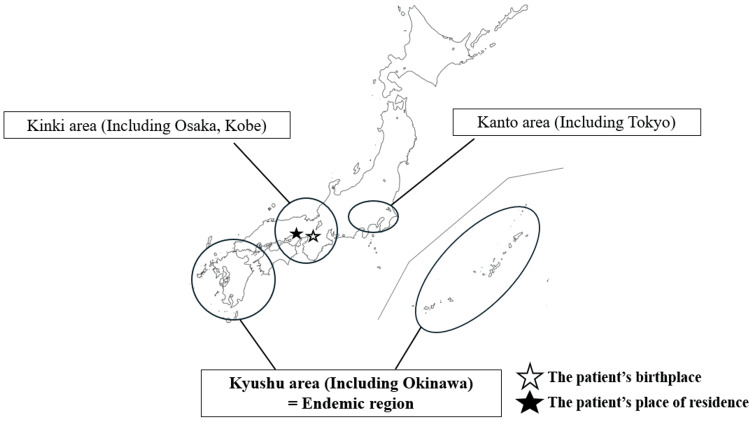
Map of Japan showing the patient’s birthplace and place of residence The patient was born in Osaka City and has been living in Kobe City. While the Kyushu region is recognized as an HTLV-1 endemic area, Osaka and Kobe are both situated outside this endemic zone. HTLV-1, human T-lymphotropic virus type 1

During the investigation, the patient’s daughter incidentally disclosed that she had tested positive for HTLV-1 during a routine prenatal checkup at the age of 24. Based on this information, we tested the patient for HTLV-1 infection using the Elecsys HTLV-I/II assay (Roche Diagnostics GmbH, Mannheim, Germany), and she also tested positive for anti-HTLV-1 antibodies. The patient, who was born and had lived in the Kansai district, tested positive for anti-HTLV-1 antibodies. Figure 5 shows a map of Japan indicating the HTLV-1 endemic regions, along with the patient’s birthplace and place of residence.

Fortunately, she did not develop adult T-cell leukemia or HAM. The levels of urea nitrogen and creatinine continued to improve gradually, and prior to hospital discharge, her urea nitrogen was 42.8 mg/dL and creatinine was 1.55 mg/dL. At a follow-up visit 12 months after discharge, there was no improvement in blood urea nitrogen and creatinine levels, which remained at 21.8 mg/dL and 1.31 mg/dL, respectively, without proteinuria (chronic kidney disease (CKD) stage G3bA1). The patient’s urinary difficulties persisted, with ongoing bacteriuria noted on urinalysis. This clinical presentation was consistent with chronic cystitis secondary to HTLV-1-related neuropathic bladder dysfunction. Since then, the patient has continued treatment for CKD and neuropathic bladder dysfunction, but her overall condition remains good.

## Discussion

Our patient presented with fever, urinary disturbances, acute abdomen, and severe AKI. Diagnostic tests confirmed APN/AFBN, and an incidental finding revealed HTLV-1 infection. Although we did not perform a urodynamic study, her chronic urinary difficulties were suggestive of a neuropathic bladder. There was no evidence of spinal cord or cerebral lesions, which are recognized causes of neuropathic bladder [11]. However, isolated neuropathic bladder has been reported in individuals with chronic HTLV-1 infection. The proposed pathogenesis involves an inflammatory response induced by HTLV-1 infection in the detrusor center located in the sacral segment of the spinal cord [8]. Given the presence of HTLV-1 antibodies and the absence of other potential causes, we attributed the neuropathic bladder in this case to chronic HTLV-1 infection. Neuropathic bladder is a known risk factor for UTI [12]. Therefore, the possibility of underlying neuropathic bladder dysfunction should be carefully evaluated in patients with APN and chronic HTLV-1 infection, even when other neurological manifestations are absent. Close follow-up is essential to monitor for potential development of paraplegia, as neuropathic bladder may represent an early manifestation of HAM.

This case posed a diagnostic challenge due to the complexity of atypical symptoms and issues associated with APN/AFBN. The patient’s symptoms, resembling an acute abdomen at initial presentation, complicated the differential diagnosis of APN/AFBN. AFBN is a focal bacterial interstitial infection of the kidney without abscess formation and is considered an intermediate disorder within the spectrum of upper UTIs, ranging from APN to renal abscess [10]. While fever, ipsilateral flank pain, and lower UTI symptoms are typical of AFBN, atypical symptoms such as peri-umbilical pain or lower quadrant pain with guarding can occur. These atypical presentations can result in misdiagnosis of other conditions, such as acute appendicitis or cholecystitis [10]. Therefore, AFBN should be considered as a differential diagnosis in patients presenting with acute abdomen.

## Conclusions

During the diagnostic workup of pyelonephritis, thoroughly investigating underlying conditions that may predispose patients to infection is essential. When neuropathic bladder is identified, obtaining a detailed medical and birth history through patient interviews is crucial. If HTLV-1 infection is suspected, screening for HTLV-1 antibodies is necessary, as neuropathic bladder can be an early manifestation of HAM.
